# The Egh16-like virulence factor TrsA of the nematode-trapping fungus *Arthrobotrys flagrans* facilitates intrusion into its host *Caenorhabditis elegans*

**DOI:** 10.1371/journal.ppat.1013370

**Published:** 2025-08-25

**Authors:** Jennifer Emser, Lena Seidler, Elma Kovačević, Kaiwei Yu, Tim Rudolf, Elke Wohlmann, Reinhard Fischer

**Affiliations:** Karlsruhe Institute of Technology, Karlsruhe, Germany; University of California Riverside, UNITED STATES OF AMERICA

## Abstract

The first physical barrier pathogenic microbes need to overcome for host colonization is the cuticle, epidermis, or skin of an animal. The nematode-trapping fungus *Arthrobotrys flagrans* is able to catch and digest nematodes like *Caenorhabditis elegans* by overcoming this physical barrier of the nematode and colonize the entire body*.* Here we characterized TrsA (trap-specific protein), a virulence factor of *A. flagrans* that is produced in the adhesive traps of the fungus and in trophic hyphae. Deletion of *trsA* increased the time until the fungus entered the nematode. Heterologous expression of *trsA* in *C. elegans* shortened its lifespan and led to softening of the animal. The protein structure of TrsA displays some characteristics of lytic polysaccharide monooxygenases (LPMOs), and TrsA indeed showed copper-dependent oxidase activity. LPMOs are enzymes with roles in the degradation of polysaccharides such as chitin and cellulose but also in organismic interactions and can be found in bacteria, fungi, plants, and insects. We hypothesize that TrsA defines a new LPMO family that likely targets oligosaccharides in the cuticle and the extracellular matrix of *C. elegans* and thereby facilitates entry into *C. elegans* and spreading of the fungus in the nematode body.

## Author summary

Filamentous fungi are often degrading dead, organic material and thereby closing the carbon cycle. However, many fungi may colonize living organisms, be it for the benefit of both partners in symbiotic interactions like the mycorrhiza or be it in pathogenic interactions where fungi can cause severe damage to the host. One very peculiar lifestyle is found in nematode-trapping fungi. These fungi can grow saprotrophically, but if they are starving and nematodes are present, they form trapping devices to capture living nematodes, penetrate the animals with growing hyphae, and subsequently colonize the entire body. The penetration and overcoming of the nematode defense require the action of lytic enzymes in combination with small-secreted proteins without enzymatic activity. Here, we characterized one such virulence or effector protein and showed that the fungi secrete the protein upon entry into the nematode body and during spreading inside the host. The protein is likely a lytic polysaccharide monooxygenase which lyses molecules of the cuticle and of the extracellular matrix to weaken the tissues and facilitate entry and colonization.

## Introduction

Pathogens are a constant threat for all living organisms, and the success of a pathogen usually reflects a fine-tuned interaction shaped over time in evolution. Especially microbial pathogens often evolved a battery of virulence factors to overcome defense mechanisms of the host. The first mechanical barrier for microbial colonization of a plant or animal host is their surface. Many pathogens circumvent this barrier by using natural openings such as stomata in the case of plants or the mouth, nose, ears, urinary or intestinal tract, or the vagina in the case of animals. Wounds are also obvious entry points for pathogens. However, some filamentous fungi can penetrate the cuticle and epidermis of plants or the skin of animals. The rice blast fungus *Magnaporthe oryzae* develops appressoria on the leaf surface from where a penetration hypha is pushed with osmotic pressure into the host cell [1]. An example of microbial penetration of an animal’s skin are nematode-trapping fungi (NTF). *Arthrobotrys flagrans* is such a fungus which is widespread in soil habitats [2]. It can switch from a saprotrophic to a predatory lifestyle in low-nutrition environments. In the presence of nematodes, it forms networks of adhesive traps that immobilize and digest nematodes. The induction of trap formation and the luring of nematodes into the traps depends on various odorants and chemical communication [3–5]. *A. flagrans* produces volatile polyketide derivatives, the arthrosporols, which inhibit trap formation in the presence of sufficient nutrients [6]. Using the same biochemical pathway, *A. flagrans* also produces 6-methyl salicylic acid at the border of the colonies to lure nematodes into the mycelium [7]. However, induction of trap formation also requires the presence of large numbers of nematodes. Nematodes are sensed through nematode-derived ascarosides, pheromones with important functions in nematode development. Ascarosides lead to the downregulation of arthrosporols, the inhibitor of trap formation. Hence, trap formation is initiated if ascaroside concentrations rise. The sensor for ascarosides in *A. flagrans* was found to be a G-protein coupled receptor [8,9]. In addition to chemical communication, mechanical sensing plays a role [9].

After a nematode is trapped, the fungus penetrates the cuticle and epidermis and forms a bulbous underneath the epidermis, from where hyphae grow out to colonize the entire nematode body. The mechanism with which NTFs overcome the robust cuticle and epidermis is still not understood. It was hypothesized that lytic enzymes are secreted to dissolve the skin, but genome-wide expression analyses revealed up-regulation of proteases especially later during predation [10,11]. However, although no distinct structure like an appressorium is formed, which could generate high pressure, pictures from the penetration suggest that pressure also plays a role during penetration [12]. Therefore, penetration likely is a combination of pushing hyphal tips plus lytic enzymes. Besides enzymes, small-secreted proteins (SSPs) without any domains indicative for certain catalytic activities, may play a role [3,13]. Such SSPs are called effector proteins and are important virulence factors in many bacterial or fungal infections. Indeed, first candidates in *A. flagrans* have been characterized with the cysteine-rich peptides NipA (nematode-induced protein A) and CyrA (cysteine-rich protein A), which are transcriptionally induced in traps [13,14]. NipA is secreted at the contact site of the penetration hypha with the nematode body and possibly interacts with the integrity of the collagen fibers in the skin. Without NipA, penetration is still possible but takes longer. CyrA acts later in the infection process, is secreted at the infection bulbus, and accelerates paralysis. The fungus in addition induces neuropeptide-like proteins in *C. elegans*, which enhance neurodegeneration and thereby also help the fungus to successfully colonize the nematode [15].

The secretome of *A. flagrans* comprises over 600 proteins, of which over 200 are classified as small-secreted proteins (SSPs) [12], suggesting a complex infection process relying on the concerted action of SSPs and lytic enzymes. Here we characterized a novel secreted protein, TrsA (trap-specific protein A), with oxidase activity. The enzyme may target carbohydrate chains in glycoproteins and belongs to the family of lytic polysaccharide monooxygenases (LPMO). Glycoproteins are found in the cuticle and in the extracellular matrix (ECM) of *C. elegans* cells. Destabilization or degradation of the cuticle and/or the ECM would facilitate the penetration and spreading process. We propose TrsA as the first member of a new LPMO family.

## Results

### *trsA* expression is upregulated during the predatory lifestyle

**Fig 1:**
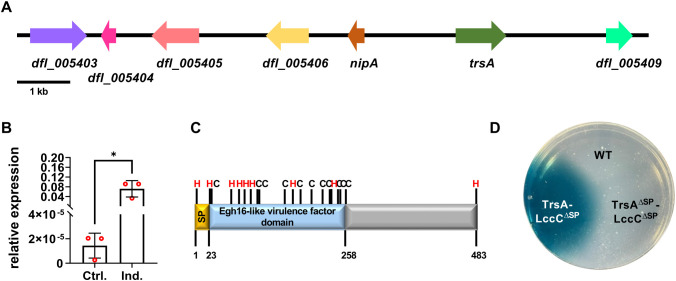
*trsA* is upregulated during infection with *A. flagrans.* **(A)** Position of *trsA* in the genome of *A. flagrans*. **(B)** Relative expression of qRT PCR shows that *trsA* is induced during infection with *A. flagrans*. RNA was extracted from vegetative (Ctrl.) and nematode-induced mycelium (Ind.). Significance was determined using the unpaired two-tailed Student’s t-test (p-value = 0.02; *). **(C)** *trsA* has a predicted signal peptide (AA 1–23; yellow) and a Egh16-like virulence factor domain (AA 24–258; light blue). The C-terminal part has no predicted domain (grey). The Egh16 domain harbors several cysteine (black) and histidine residues (red). **(D)** Laccase assay to proof the functionality of the SP. Full-length *trsA* was fused to lccC^∆SP^ gene of *A. nidulans* and expressed under the constitutive promoter *gpdA*. Transformants were incubated on PDA plates with 2 mM ABTS for 2 days at 28°C. The laccase oxidizes ABTS when secreted, which leads to a color change. The assay shows that the SP of *trsA* is functional. Lack of the signal peptide of TrsA prevented secretion (*trsA*^∆SP^).

The stage-specific expression of genes can be a first hint to identify potential virulence genes. One example is NipA, which is expressed and secreted prior to penetration [14]. The analysis of the genomic context of *nipA* revealed the presence of a gene encoding a putative Egh16-domain-containing protein (Fig 1A). *egh16* and *egh7* are genes that were first identified in the biotroph *Erysiphe graminis f.sp. hordei* as differentially expressed in germinating conidia, but later they were found in different pathogenic fungi [16,17]. Because some Egh16-domain-containing proteins play roles in pathogenic interactions, the presence of two genes with putative roles in pathogenicity in close proximity suggested the presence of a virulence island. Therefore, we analyzed the expression of some around *nipA* (*dfl_005403*, *dfl_005405*, *dfl_005406*, and *dfl_005408*). RNA was isolated from vegetative (Ctrl.) and nematode-induced mycelium (Ind.). The expression values were normalized to the *A. flagrans* actin gene (*dfl_002353*). In addition to *nipA*, only *dfl_005408* showed strong transcriptional upregulation with a relative expression of 0.072 (± 0.034 SD; n = 3). Significance was determined using the unpaired two-tailed Student’s t-test (p-value = 0.02; *) (Figs 1B and S1). Hence, *dfl_005408* was a promising candidate virulence factor. Because it was upregulated in traps, we named it trap-specific protein A (*trsA*).

Bioinformatic analysis of the amino acid sequence of TrsA using SignalP6.0 identified a signal peptide sequence in the first 23 amino acids (SP) of the N-terminus, but TrsA does not belong to the group of SSPs, considering the length of 483 amino acids. Using InterProScan, the Egh16 domain of TrsA was determined in the 235 amino acids following the signal peptide (Fig 1C). TrsA displayed homologies to proteins with an Egh16 virulence-factor domain, but the homology was restricted to the conserved domain. TrsA orthologues were well conserved (also outside the Egh16 domain) in other NTF, such as *A. oligospora* but not in other fungal saprotrophs or pathogens. Hence, TrsA appears to be specifically required for the colonization of living nematodes.

### TrsA is transcriptionally upregulated near and in traps and in trophic hyphae

The functionality of the SP was proven using a laccase assay. For this purpose, the full-length protein was fused to the N-terminal part of the *A. nidulans laccase C* gene without SP and expressed under the constitutive *A. nidulans gpdA* promoter. Transformants were inoculated on PDA plates with 2 mM ABTS and incubated for 2 days at 28°C. Laccase was apparently secreted, because ABTS in the medium was oxidized as indicated by a blue color. As a control, TrsA without SP was fused to laccase. In this case, no color change was observed (Fig 1D).

**Fig 2:**
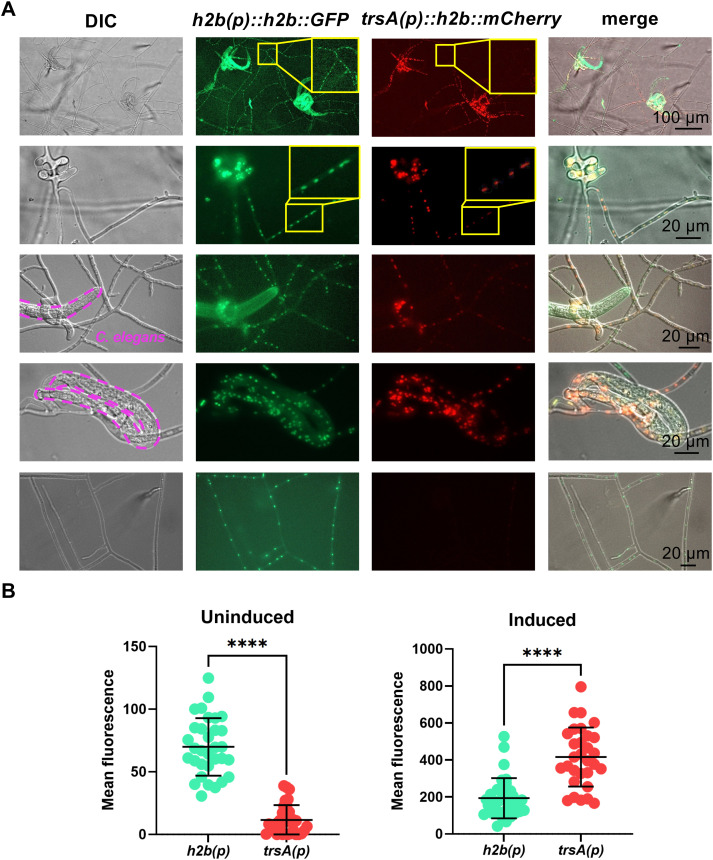
The *trsA* gene is upregulated near and in traps and in hyphae inside *C. elegans.* **(A)** The promoter of *trsA* was fused to the *h2b* gene and mCherry. As control *h2b* with its native promoter was fused to *GFP*. The GFP signal was equally intense in all nuclei while the mCherry signal was stronger in the area around the traps, in traps and in trophic hyphae. The shape of the captured nematode is indicated by a dashed line in the DIC pictures. **(B)** Quantification of the mean fluorescence of nuclei in uninduced and induced mycelium. Significance was determined using the unpaired two-tailed Student’s t-test (p-value = 0.0001; ****).

Because neither this assay nor the qRT-PCR assay allows spatial resolution of gene expression, a promoter reporter assay was performed in which the 1.4 kb sequence upstream of the *trsA*-start codon was cloned in front of an *h2b*-mCherry reporter construct. The nuclear localization signal (NLS) of the H2B protein targets the fusion protein into nuclei. Hence, nuclei are ”lighting up” if the promoter is active. The expression was compared to a constitutively expressed H2B-GFP construct. The mCherry signal was more intense near the traps and in trophic hyphae inside *C. elegans*, whereas the GFP signal was equally intense in all nuclei (Fig 2). Vegetative hyphae only showed the GFP fluorescence. The increased gene expression in traps and trophic hyphae confirmed a putative role for TrsA during infection of *C. elegans*.

### TrsA is secreted at fungal-nematode contact sites

**Fig 3:**
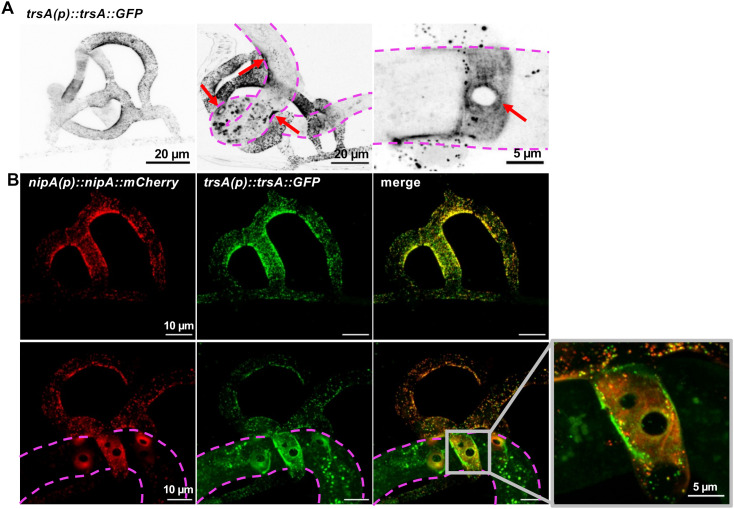
Co-localization of TrsA and NipA. **(A)** Localization of TrsA fused to GFP. TrsA localizes to the inner rim of empty traps (left), at the hyphal-nematode contact sites (red arrows, middle picture) and also in hyphae growing inside the nematode. Enlargement of a contact site (right picture). The contour of the captured nematode is indicated by a dashed line. **(B)** Co-localization of NipA and TrsA. NipA was fused to mCherry and TrsA to GFP. The localizations largely overlap in the trap. TrsA appears to be distributed in a broader area and is also additionally present in trophic hyphae inside of trapped nematodes in vesicle-like structures. NipA is not found in trophic hyphae.

Next, we aimed at localizing the TrsA protein during the predatory phase. For this, *trsA* was fused to GFP driven from its native promoter and transformed into *A. flagrans*. In infection assays we observed GFP fluorescence at the inner side of the trap, which is normally the contact site to the nematode (Fig 3A). The fluorescence signal suggested that the protein localizes to secretion vesicles, which is in agreement with the presence of a signal peptide. Co-localization with NipA fused to mCherry showed a similar distribution of the protein, although TrsA was distributed in a broader area than NipA (Fig 3B). Additionally, TrsA-GFP was visible in the trophic hyphae inside of trapped nematodes in vesicle-like structures, which clearly distinguishes it from NipA, which is restricted to the outside contact site [14]. The protein expression data suggests roles for TrsA early during the infection process but also inside the nematode. This is in agreement with the function of other Egh16-domain-containing proteins, which often play a role in early infection stages, but also points to overlapping or different functions in later stages of prey colonization [18].

### TrsA facilitates fungal penetration of *C. elegans*

**Fig 4:**
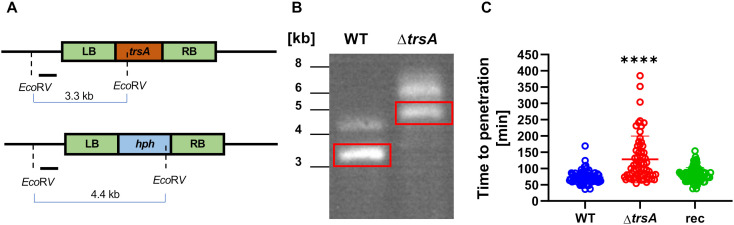
TrsA facilitates penetration. **(A)** The scheme shows the *trsA* genomic locus before (up) and after (down) integration of the construct. **(B)** The deletion was confirmed with a Southern blot. Genomic DNA was digested with *Eco*RV, and the blot hybridized to a DIG-labeled probe derived from the 5’-region indicated by a line in (A). The red boxes indicate the expected bands. **(C)** Penetration assays of the *trsA*-deletion strain show a significant increase of time until the nematode is penetrated compared to the WT and a re-complemented (rec) strain (unpaired two-sided students t-test; p-value < 0.0001, ****). All data points (technical and biological replicates) are displayed.

The expression of TrsA at the contact site with the nematode points towards a function in penetration, whereas the expression in trophic hyphae points towards functions during hyphal spreading in the body or in the digestion process. Ultimately, TrsA may target the same molecular process, and the question is what penetration and hyphal spreading, or digestion, have in common. To address these questions, we created a *trsA*-deletion strain by replacing *trsA* with a hygromycin resistance cassette via homologous recombination (Fig 4A). The deletion event was confirmed by Southern blot and PCR (Figs 4B and S2). The transgenic strain produced the same amounts of traps as wild type. Next, we performed infection assays. The time from the moment a nematode was trapped until it was penetrated was measured. As a marker for successful penetration, we used CyrA-mCherry localization in the infection bulb. CyrA is the first effector described in *A. flagrans* and appears at the infection bulb as soon as it is formed [13]. The deletion strain required a significantly longer time until the nematode was penetrated compared to the wild type (Fig 4C). Re-complementation of the *trsA*-deletion strain with a wild-type copy restored full virulence, with a similar penetration time as wild type. This confirms that *trsA* is involved in the penetration process. The spreading dynamics of the mycelium inside the nematode body was not obviously different from the wild type, although a good marker is missing for exact measurements.

### TrsA is a copper-dependent oxidase

**Fig 5:**
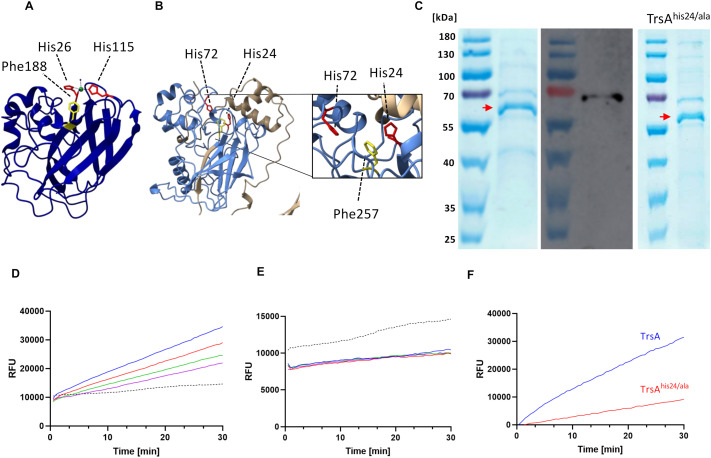
TrsA has oxidase activity. **(A)** Protein structure of the *Pl*AA10 LPMO from *Photorhabdus luminescens* [20]. The core structure of LPMOs consist of two β-sheets with 7 or 8 antiparallel strands. His26 and His115 of *Pl*AA10 form the histidine brace (red) which together with Phe188 (yellow) bind copper (green) for enzyme activity. **(B)** Putative structure of the core of TrsA (amino acid 1-265 plus 401-485) without the region (266-400), which had only very low confidence. TrsA shares similarities with LPMOs such as two β-sheets with 8 antiparallel strands and two histidines that could potentially form the histidine brace (His24 and His72; red). Together with Phe257 (yellow) it could form the active site. The putative His brace lies in the Egh16 domain (light blue). **(C)** SDS-PAGE and Western blot after purification of TrsA and TrsA^his24/ala^. The red arrow indicates TrsA, which has a molecular mass of 53 kDa. **(D)** LPMO assay of the purified TrsA that was incubated in CuCl_2_. Reduced fluorescein is oxidized to fluorescein by TrsA which leads to a steadily increased fluorescence over time. The fluorescence correlates with the protein concentration (blue: 1 µM; red: 0.75 µM; green: 0.5 µM; purple: 0.25 µM). Elution buffer without protein was used as control (black). **(E)** LPMO assay of TrsA without prior incubation in CuCl_2_. The fluorescence of all concentration is lower than the control and there is no difference between the concentration. Color code as in (D). **(F)** LPMO assay of 1 µM TrsA and 1 µM TrsA^his24/ala^. TrsA^his24/ala^ shows reduced activity compared to wild-type TrsA. The RFU values were normalized to the buffer.

Egh16-like proteins and lytic polysaccharide monooxygenases (LPMOs) are distantly related proteins [19]. Characteristics associated with LPMOs are a core of two β-sheets consisting of 7 or 8 antiparallel strands and two histidines that form a so-called *histidine brace,* as seen in the *Pl*AA10 LPMO from *Photorhabdus luminescens* (Fig 5A) [20]. The first histidine is usually located very close to the N-terminus shortly after the signal peptide, whereas the position of the second one varies and locates further downstream in the sequence. The histidine brace, often together with phenylalanine or tyrosine, binds a copper ion, which is essential for enzyme activity. In the sequence of TrsA, the histidine close to the N-terminus appears to be conserved as compared to LPMOs, and several other histidines can be found downstream from the first histidine. Although sequence comparisons with TrsA revealed that it is only distantly related to LPMOs, the modeled three-dimensional structure of TrsA revealed similarities to LPMOs with a β-sandwich core with 8 antiparallel strands and two histidines in the Egh16-like domain (His24 and His72) that could form a histidine brace (Fig 5B). Additionally, there is phenylalanine 257 facing these two histidines, which is essential for the active site in some LPMOs. Hence, structural predictions suggested enzymatic activity of TrsA.

To test this hypothesis, we aimed at testing such activity. The substrates for LPMOs are usually polysaccharides like chitin or cellulose, which are broken down by oxidative cleavage of the β-(1,4)-linked bonds. However, LPMOs are also able to oxidize reduced fluorescein because the central carbon has a C-H bond that can be cleaved in a reaction that resembles the deprotonation by an LPMO [21]. This fluorescein-based LPMO assay was performed with the TrsA protein expressed and purified from *E. coli*. TrsA without the SP was fused with a Cytiva Protein Select tag at the N-terminus and a 6x His tag at the C-terminal end. The identity of the expressed tagged protein was confirmed by SDS-PAGE and Western blotting using anti-His-tag antibodies (Fig 5C). The Cytiva Protein Select tag is autocatalytically cleaved off after purification, which results in a protein without an N-terminal tag. However, during expression in *E. coli* the N-terminal tag interferes with the first histidine residue required for copper insertion. Therefore, copper can only be incorporated in the putative histidine brace after purification and self-cleavage of the tag. For this, the protein was incubated in CuCl_2_, and unbound copper was removed with a desalting column. To test the importance of the histidine residue 24, which was predicted to form the histidine brace, we mutated the histidine to an alanine and also purified the protein from *E. coli* (Fig 5C).

In the following enzyme assay, reduced fluorescein was used as substrate, whose fluorescence increases upon oxidation. In the presence of TrsA, fluorescein was indeed oxidized over time, and the enzyme activity was protein-concentration dependent (Fig 5D). As a negative control, elution buffer without protein was used. TrsA protein that was not incubated in CuCl_2_ for copper incorporation did not result in any activity (Fig 5E). The mutated TrsA^his24/ala^ protein had much lower enzyme activity (Fig 5F). Taken together, we present first experimental evidence that the Egh16-containing TrsA protein probably has LPMO activity.

After the observation of the enzyme activity of TrsA we tested if the addition of purified TrsA to live *C. elegans* would affect the animals. However, we did not observe any alteration of the morphology or behavior of the nematodes.

### TrsA expression in *C. elegans* causes morphology defects

**Fig 6:**
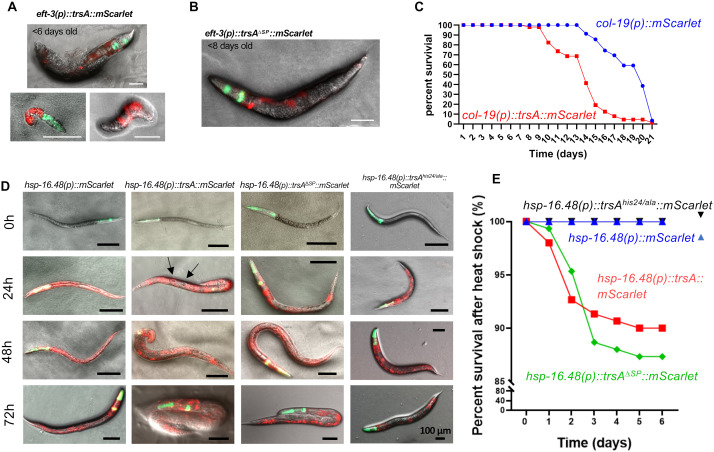
Heterologous expression of *trsA* in *C. elegans* causes severe morphological alterations. **(A)** Expression of *trsA* fused to *mScarlet* with the all-tissue promoter *eft-3* with SP and **(B)** without SP. The construct resulted in mosaic expression in the nematodes, and some exhibited abnormal phenotypes like bulky extrusions. No stable F2 generation could be obtained with either construct. Scale bars, 50 µm. **(C)** Survival rate in percent of *C. elegans* expressing *trsA* fused to *mScarlet* under the epidermal *col-19* promoter compared to the control expressing *mScarlet* using the same promoter. The nematodes expressing *trsA* show a lower survival rate from day 10 on, with a strong decrease after day 13. **(D)** *C. elegans* expressing *trsA* under the heat shock promoter *hsp-16.48*. Pictures were taken 0, 24, 48 and 72 hours after the heat shock (37°C for 1 hour). 24 h post heat shock, some nematodes expressing *trsA* exhibited morphological abnormalities like a softened body surface or bubble-like structures (arrows). The phenotypes of the strain expressing *trsA*^*∆SP*^ were milder. The control that only expresses *mScarlet* as well as the strain expressing *trsA*^*his24/ala*^ did not show morphological changes. **(E)** Survival rate in percent of *C. elegans* expressing *trsA, trsA*^*his24/ala*^ and *trsA*^*∆SP*^ under the heat-shock promoter *hsp-16.48* fused to *mScarlet*. The strain expressing *trsA* shows a decreased survival rate compared to the control.

The action of the secreted TrsA protein is restricted to the contact site of the hypha with the nematode body or very locally during the colonization. Therefore, direct effects are usually difficult to see. A great method to amplify the effects of virulence factors is their heterologous expression in the host organisms [14]. We first expressed *trsA* as an extrachromosomal array under the all-tissue promoter *eft-3*, resulting in mosaic expression across all developmental stages and tissues of the nematode. *trsA* was expressed both with and without signal peptide and was C-terminally fused to mScarlet. For selection of positive transformants, nematodes were co-injected with a green-fluorescent pharyngeal marker (*myo-2(p)::GFP*). A total of 24 injections were performed for each construct. From the injections with full-length TrsA, we obtained 29 transformants from 8 injected nematodes. Five of the F1 transformants exhibited abnormal body phenotypes, including bulky deformations or extrusions, and one line did not develop beyond the L2 stage (Fig 6A). Only one line produced an F2 generation, which eventually lost the mScarlet signal and was therefore discarded. For the construct without signal peptide, 19 lines were obtained from six injections, with four F1 transformants displaying an abnormal phenotype (Fig 6B). No stable F2 generations could be obtained from either construct, suggesting deleterious effects of TrsA.

To test if the reduced cysteines play a crucial role for the function of TrsA, like they do for NipA, we mutated cysteines at positions 25, 86 and 142. Those three cysteines were predicted not to be involved in disulfide bridges. Mutation to alanine should also not alter the secondary structure of the protein, as predicted by AlphaFold 3. The gene fragment was C-terminally fused to mScarlet, and expression in the nematode was driven by the *eft-3* all-tissue promoter. 12 injections were performed, from which 5 produced transformed progeny from where 23 lines could be obtained. As for the expression of the native protein, we were not able to maintain an F2 generation with mScarlet signal. From 23 lines, 4 showed a strong mScarlet signal throughout the whole body but did not develop over the L2 stage. Only worms with weak or no mScarlet signal could form F2 generations. Among these lines some offspring with strong mScarlet signal developed abnormal body phenotypes, like nematodes expressing the native TrsA protein, indicating that the mutation of reduced cysteines to alanine did not have an effect on the functionality of TrsA in the nematode.

### TrsA expressed in *C. elegans* reduces its lifespan

To further investigate the impact of TrsA on the physiological functions of *C. elegans*, we expressed TrsA under the epidermal promoter *col-19* in *C. elegans* and performed a lifespan analysis. Compared to the control group (expressing mScarlet under the *col-19* promoter), the *trsA*-expressing strain exhibited a significant decline in survival rates on days 10 and 13 (Fig 6C). Notably, beginning on day 9, the survival rate of the *trsA*-expressing group remained consistently lower than that of the control group; this trend persisted until the end of the assay. Severe morphological phenotypes were not observed as compared to the expression with the *eft-3* all tissue promoter.

To assess the kinetics of TrsA activity, we further utilized a heat shock promoter to drive *trsA* expression with and without SP as well as *trsA* with mutated His24, which is part of the putative *his brace,* and monitored phenotypic alterations following heat shock treatment. Initially, the nematodes were synchronized to the L1 stage and incubated for 24 hours to allow larval development to the L3 stage prior to the heat shock. The heat shock was performed for 1 hour at 37°C. Phenotypes were then monitored at 24, 48, and 72 hours post-heat shock (Fig 6D). At 24 hours post-heat shock, nematodes expressing *trsA* already exhibited noticeable morphological abnormalities, primarily characterized by a softening of the body surface and the emergence of bubble-like structures. It was very difficult to transfer such “softened” nematodes to fresh plates in comparison to wild type (S1 and S2 Movies). In contrast, the phenotype was milder when TrsA was expressed without the signal peptide. The nematodes expressing TrsA with mutated His24 did not show abnormalities. At 48 and 72 hours post-heat shock, the morphological phenotypes in the *trsA* group became even more pronounced, whereas the *trsA*^*ΔSP*^ group continued to display comparatively subtle changes. (Fig 6D). The nematodes of the trsA^*his24/ala*^ group still did not show alterations after 72 hours. Since the nematodes expressing *trsA* and *trsA*^*∆SP*^ showed morphological changes, the survival rates of these strains after heat shock were analyzed. They showed a significant decline in viability starting one day after heat shock (Fig 6E), with the trend stabilizing by day 3, ultimately resulting in an overall lower survival rate compared to the control group. TrsA without the signal peptide appeared to be as toxic as secreted TrsA when comparing the lifespan. This may be due to the oxidase activity of TrsA in the cells. Induction of TrsA^his24/ala^ did not affect the survival rate.

In summary, TrsA expression severely affects the morphology and fitness of *C. elegans*, and His24 plays a critical role in TrsA activity.

## Discussion

**Fig 7:**
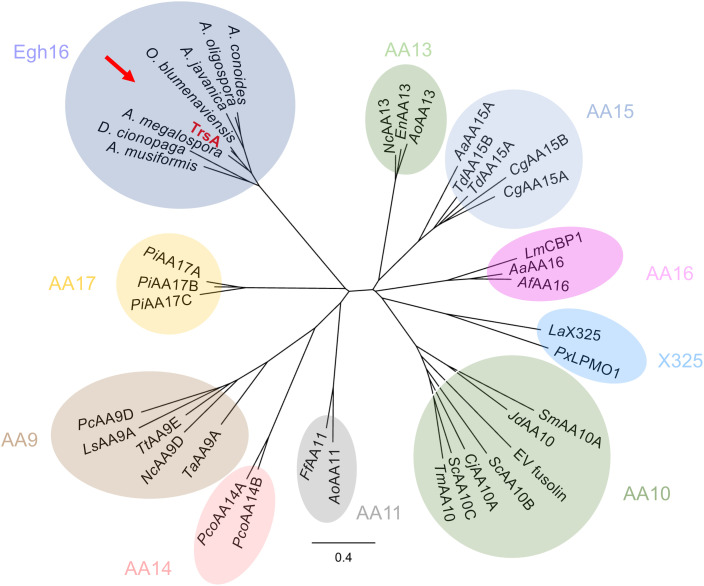
TrsA defines a new LPMO family. Phylogenetic tree construction was based on the same proteins used before, representing eight families [22]. X325 defines a family with LPMO-like proteins. TrsA (red) and its orthologs were included in the LPMO family tree. Orthologs of TrsA were only found in other NTFs. We propose that TrsA belongs to a new LPMO family. Accession numbers for the orthologs are as follows: *A. musiformis* [KAK6509352.1], *D. cionopaga* [KAF3932045.1], *A. megalospora* [KAK6517482.1], *O. blumenaviensis* [KAK6349351.1], *A. javanica* [KAK6351920.1], *A. oligospora* [KAF3175641.1] and *A. conoides* [KAK6499398.1]. The tree was created with Geneious Prime 2025.1.1. The sequences were aligned with Clustal Omega, and the tree was calculated with Jukes-Cantor and Neighbor-Joining.

Lytic polysaccharide monooxygenases (LPMOs) have been discovered as auxiliary enzymes for the degradation of recalcitrant and rather inert polysaccharides such as plant cell wall polysaccharides or chitin [22,23]. After the oxidative cleavage of glycosidic bonds, the substrates become accessible to canonical hydrolases. Eight different families of such enzymes with auxiliary activity (AA) have been described in the CAZy (Carbohydrate active enzymes) database (Fig 7). Meanwhile, there are examples of LPMOs having moonlight functions besides biomass degradation [22]. With the new virulence factor TrsA, we characterized the first Egh16-domain-containing protein that shows characteristics of an LPMO, although Egh16-like proteins are only distantly related to LPMOs based on sequence comparisons. We show that TrsA catalyzes the oxidation of fluorescein, a first indication for TrsA being an LPMO. TrsA is conserved in several other nematode-trapping fungi (NTF), where the protein also contains an Egh16 domain and several histidines that may form a histidine brace. These NTF species all form adhesive trapping networks or adhesive nobs. The protein was not found in NTF with other trapping devices like constricting rings. It was also not found in other saprotrophs or pathogens. Therefore, we propose that the LPMOs of such NTFs define a new family of LPMOs (Fig 7). It will be a challenge for future research to discover the natural substrate(s) for this new enzyme family.

Here we are going to discuss different possibilities for how such an enzyme activity could be auxiliary for *C. elegans* penetration and colonization. We envision three different scenarios: (i) TrsA as an LPMO of cuticle or ECM glycoproteins, (ii) TrsA as a chitin-active LPMO that helps to restructure the cell wall during the formation of the penetration peg, and (iii) TrsA as an LPMO that contributes to overall virulence.

The cuticle of *C. elegans* is mainly comprised of crosslinked collagen. The outer layer, called the epicuticle, is rich in lipids and overlaid by a coat of glycoproteins [24]. The carbohydrate chains of the glycoproteins could be the target of TrsA, and breakdown of these carbohydrates could facilitate the penetration of the nematode. In addition, cell-cell contacts rely on polysaccharides and glycoproteins in the extracellular matrix, which also could be a target of TrsA. For instance, downregulation of laminin subunits leads to embryonic lethality, where embryos appear very disorganized and fail to form proper tissues and organs [25]. This would explain the “soft” phenotype when TrsA is heterologously expressed in *C. elegans*.

The second proposed possibility for a function of TrsA is based on protein structure comparison using DALI (http://ekhidna2.biocenter.helsinki.fi/dali/), which revealed similarities to LPMOs that cleave chitin or act as chitin-binding proteins. If chitin is the substrate for TrsA, it might be necessary on the fungal side during the formation of the penetration peg and growth of trophic hyphae. TrsA could be involved in restructuring the fungal cell wall to prepare it for nematode penetration or for growth in the special intra-nematode environment. Likewise, in plant pathogens, Egh16-like proteins are known to be necessary during the formation and penetration of the appressorium [26]. There are also other proteins with an Egh16-like domain encoded in the genome of *A. flagrans* that are predicted to be chitin-binding type 4 proteins. Such an activity could explain the slower penetration of *A. flagrans* into *C. elegans,* but how would that explain the deleterious phenotypes after heterologous expression in the nematode? To explain this, one has to consider that the pharynx and the eggshells of *C. elegans* contain chitin. The latter might explain why some transgenic lines did not have any offspring. However, the severe morphological alterations are probably difficult to explain with effects on the pharyngeal apparatus.

The function could also be completely different from the degradation of oligo- or polysaccharides. There are several reports of LPMOs contributing to virulence or enabling a pathogen to hide from the immune system of the host [22]. There is a chitin-cleaving LPMO in *Pseudomonas aeruginosa*, CbpD (chitin-binding protein D), an important virulence factor during infection of humans or mice [27]. Even though neither mammals nor *P. aeruginosa* contain chitin, the protein causes changes in the proteome. CbpD also promotes resistance to the immune system of mice and humans. Although such an effect of TrsA on the innate immune system of *C. elegans* is currently not supported by any experimental evidence, one should keep this possibility in mind and be open to new functions of this interesting class of LPMOs beyond polysaccharide degradation.

Taken together, the most likely function of TrsA appears to be the destabilization of the cuticle and cell-cell contacts through glycosidic-bond oxidation. It thereby would define a new family within the LPMOs.

## Methods

### Strains and culture conditions

*A. flagrans* (CBS 349.94) was obtained from the CBS-KNAW culture collection (The Netherlands) and was cultured at 28°C on potato dextrose agar (PDA). *C. elegans* cultivation and synchronization was performed according to the WormBook [28]. Strains are listed in S1 Table.

### Trap induction of *A. flagrans*

For trap induction spores of *A. flagrans* were inoculated on thin low-nutrient-agar slides (KCL 1 g/L, MgSO_4_-7H_2_O 0.2 g/L, MnSO_4_-4H_2_O 0.4 mg/L, ZnSO_4_-7H_2_O 0.88 mg/L, FeCl_3_-6H_2_O mg/L, Agar 15 g/L, pH 5.5) and co-incubated with *C. elegans* at 28°C in darkness for at least 12 hours [12].

### Generation of transgenic microorganisms

Protoplast transformation of *A. flagrans*: Protoplast transformation was carried out as described [12]. 6x10^6^ protoplasts were transformed with 6–7 µg of DNA. Transformants were incubated at 28°C for 4–7 days on PDA supplemented with 100 µg/ml hygromycin-B or 150 µg/ml geneticin (G418) or on plates containing both 150 µg/ml geneticin and 100 µg/ml nourseothricin.

Microinjection of *C. elegans*: To generate transgenic *C. elegans* strains expressing extrachromosomal arrays, a DNA mix of 5 ng/µl linearized plasmid harboring the desired transgene, 5 ng/µl pharyngal marker plasmid and 140 ng/µl 1 kb DNA Ladder (Eurofins) as filler, was injected into the gonads of young adult worms. Co-injection marker positive transformants were selected.

### Plasmid construction

All plasmids are listed in S2 Table and oligonucleotides in S3 Table. For the laccase-assay, the modified vector pOF018 was used for the expression of the *A. nidulans lccC* gene (AspGD identification AN5397) under the constitutive *A. nidulans* glyceraldehyde-3-phosphate dehydrogenase (*gpdA*) promoter as described [12]. The *trsA* gene sequence was fused to the 3’ end of the laccase C by insertion into the vector backbone using the restriction enzymes *Asc*I and *Age*I. The control plasmid was built using Gibson assembly. Standard protocols were used for *E. coli* transformation and plasmid isolation (https://www.cshlpress.com/pdf/sample/2013/MC4/MC4FM.pdf) [29].

For the promoter fusion 1.4 kb upstream of the ORF of *dfl_005408* were amplified with overhangs in the pVW23 vector. The backbone was amplified using primers binding outside of the *h2b* promoter. Both amplicons were assembled in a 3:1 ratio using Gibson assembly.

Plasmids for TrsA localization were constructed using the Gibson assembly method. The *trsA* promoter was amplified together with the *trsA* ORF with gDNA as template and cloned into the pJM01 vector.

For heterologous expression in *C. elegans* the open reading frame of *trsA* was amplified with or without signal peptide (AA 1–23) from cDNA and cloned into a modified version of the pPF37 vector using Gibson assembly. For the localization of the constructs TrsA was tagged C-terminally with *mScarlet*. The constructs were expressed under the all-tissue *eft-3* promoter or the hypodermal *col-19* promoter. For inducible expression *trsA* was cloned under heat inducible promoter *hsp-16.48*.

### Laccase assay

The Laccase-assay was performed as described [12,30]. Mycelium was transferred to a LNA plate containing 2 mM ABTS using a toothpick. Plates were incubated for 48 h at 28°C. Laccase activity was correlated with blue ABTS• formation.

### Southern hybridization

To verify the *trsA* deletion, Southern blot analysis was conducted. DNA from *trsA*-deletion mutants and wild-type strains was isolated and digested overnight with *Eco*RV (New England Biolab, Frankfurt). The analysis followed standard protocols. A labeling probe was generated by PCR amplification of the *trsA* 5’ flanking site using specific primers and DIG-labeled dNTPs (Roche, Mannheim). The DNA was transferred to a nylon membrane (Roti-Nylon plus, Roth, Karlsruhe) and incubated with a 1:10,000 dilution of Anti-Digoxigenin-AP Fab Fragment (REF 11093274910; Roche, Mannheim). To develop the membrane, CDP-Star solution (Roche, Mannheim) was added just before exposure in a Chemi-Smart chemiluminescence system (Peqlab).

### RNA extraction and quantitative RT-PCR

For RNA extraction, 10^6^ *A. flagrans* spores were plated on cellophane-covered LNA plates and incubated at 28°C for 48 hours to obtain uninduced samples. To prepare induced samples, a mixed population of *C. elegans* was added after the first 24 hours, and co-cultivation continued for another 24 hours at 28°C. Trap formation was observed microscopically, after which the mycelium was collected and immediately frozen in liquid nitrogen. The material was ground using a micro pestle. Total RNA was isolated with Trizol reagent (Invitrogen, Karlsruhe), and DNase digestion was performed with the Turbo DNA-free Kit (Invitrogen, Karlsruhe). qRT-PCR was carried out using the Luna Universal One-Step RT-qPCR Kit (NEB) on a CFX Connect Real-Time PCR Detection System (Bio-Rad, Munich). Each reaction mix contained 0.2 μM oligonucleotides and 100 ng of RNA in a total volume of 20 μl. The gamma actin orthologue DFL_002353 was used as the internal reference gene. Significance was calculated using the unpaired *student’s t-test*.

### Virulence assay

To investigate the impact of the *trsA* deletion on trap formation, nematode capture behavior, and nematode penetration, spores of the designated strains were transferred to thin LNA slides. Trap formation was induced by co-incubation with a mixed population of *C. elegans*. After 24 h, trap formation was observed microscopically, areas with traps were marked and non-captured nematodes were gently washed off with dH_2_O. Long-term recordings were made using a Zeiss AxioObserver.Z1 microscope equipped with a multi-laser module, a spinning disk module (CSU-X1M 5000), and an AxioCam MRm camera. Several positions with traps were saved and a synchronized population of young adult N2 nematodes was added just before the start of the recording. The experiment was set to run for 11 h, capturing images approximately every 11 minutes in the DIC and RFP channels. Recordings were analyzed for nematodes escaping the trap networks and for penetration time. The penetration time was measured from the first contact of the nematode with the trap until the accumulation of CyrA-mCherry. The experiments were repeated in biological and technical replicates. The statistical significance was calculated using the student’s t- test.

### Lifespan assay

Synchronized L4-Larvae were transferred to NGM-Plates with OP50 lawn containing 50 mM Fudr (5-Fluor-2’-Desoxyuridin) to suppress formation of offspring. Day 0 was considered the day of transfer to the Fudr plates. Alive nematodes were counted by movement. If no movement was observed, the nematode was gently touched using a platinum wire and observed for movement for several seconds. If no movement was observable the body was removed from the plate and counted as dead. Nematodes showing vulval protrusion were removed from the plates and censored. The obtained data was used to plot a survival curve using the online tool OASIS [31]. The experiments were repeated in biological triplicates with technical replicates. After the first biological replicate the experimenter was blinded to the experimental group.

### Protein domain prediction and AlphaFold 3

The protein sequence of TrsA (RVD87167.1) was obtained from the NCBI protein database. SignalP 6.0 [32] was used for signal peptide prediction and protein domains were predicted using InterProScan [33]. TrsA was modeled using AlphaFold 3 via the AlphaFold Server [34]. Protein structures were visualized with ChimeraX [35].

### Phylogenetic tree

The phylogenetic tree was created with Geneious Prime 2025.1.1. Sequences of known LPMO proteins were taken from [22] and were aligned using Clustal Omega. The tree was calculated with Jukes-Cantor and Neighbor-joining. Accession numbers for the TrsA orthologs are: *A. musiformis* [KAK6509352.1], *D. cionopaga* [KAF3932045.1], *A. megalospora* [KAK6517482.1], *O. blumenaviensis* [KAK6349351.1], *A. javanica* [KAK6351920.1], *A. oligospora* [KAF3175641.1] and *A. conoides* [KAK6499398.1].

### Protein purification

For protein purification the Cytiva Protein Select tag was cloned n-terminal to TrsA into the pET28a vector (Novagen). It contains an Isopropyl β-D-1-thiogalactopyranoside (IPTG) inducible T7 promoter, kanamycin resistance and a C-terminal 6x His-tag. The plasmid was transformed into the *E. coli* strain BL21 DE3 and grown in 2 l liquid LB medium supplemented with 100 mM sorbitol, 2.5 mM betaine and 50 µg/ml kanamycin. The culture was incubated shaking at 180 rpm at 37°C until an OD_600_ of 0.6. To induce the expression of TrsA, 0.5 mM IPTG was added to the culture after cooling down and was incubated at 15°C for 16 hours. The culture was centrifuged at 9000 rpm at 4°C for 15 min. The supernatant was discarded, and the pellet was resuspended in 20 ml buffer A (50 mM Tris-HCL, 300 mM NaCl, 0.05% Tween20, 10% glycerol, 1 mM PMSF, pH = 7.8). The cells were disrupted with a high-pressure homogenizer (Emulsiflex-C3) and centrifuged at 18,000 rpm at 4°C for 45 min to separate the pellet. For protein purification, the ÄktaPure (Cytiva) was used with a HiTrap Protein Select column as instructed by the manufacturer. The protein was eluted with buffer A. Purified TrsA was incubated in 3X excess CuCl_2_ for 1 hour on ice and unbound CuCl_2_ was removed with a PD-10 desalting column (Cytiva). A Vivaspin ultrafiltration unit (Sartorius) with 10,000 MWCO was used to concentrate the protein. SDS-PAGE was performed following standard protocols using the PageRuler 180 kDa prestained protein ladder (Thermo Fisher Scientific). The gel was stained with Roti Blue quick (Carl Roth, Karlsruhe). Western blot was performed after standard protocols using Anti-His antibody dilution of 1:3,000 and Anti-mouse-IgG HRP antibody dilution of 1:15,000 (Sigma Aldrich).

### LPMO assay

The LPMO assay was performed as described [21].

### Microscopy

For microscopy, spores were transferred onto thin LNA slides using a sterile toothpick and incubated at 28°C for at least 12 hours with a mixed population of C. elegans to induce transformation. For epi-fluorescence microscopy and differential interference contrast (DIC) images, a Zeiss AxioImager.Z1 was used with the following objectives: Plan-Apochromat 63x/1.4 oil immersion, EC Plan-Neofluar 40x/0.75, 20x/0.50, and EC Plan-Neofluar 10x/0.30. Detection was carried out with an MRm camera using Zen Blue Software (2012). For high-resolution confocal microscopy, a Zeiss LSM 900 with an AiryScan 2 detector was used. For work with *C. elegans*, a Zeiss SteREO Discovery.V12 and a Zeiss SteREO Lumar.V1 with fluorescence filters were employed. For long-term recordings, a Zeiss AxioObserver.Z1 equipped with a Zeiss Multi-Laser Module (488 nm diode laser and 561 nm OPSL laser), a spinning disk module CSU-X1M 5000 and an AxioCam MRm was used.

## Supporting information

S1 Fig RT-qPCR of the neighboring genes of *trsA.* RNA was extracted from vegetative and nematode-induced mycelium. The expression was normalized to the *A. flagrans* actin gene (*dfl_002353*) **(A)** *nipA* (*dfl_005407*) is significantly upregulated in induced mycelium. Significance was determined using the unpaired two-tailed Student’s t-test (p-value = 0.0001; ****). **(B)** *dfl_005403*, *dfl_005405* and *dfl_005406* show no significant upregulation in nematode-induced mycelium.
(S1_Fig.PDF)

S2 Fig PCR confirmation of the *trsA* deletion. **(A)** The upstream to downstream region of *trsA* was amplified in the wild type (2.1 kb) and the *∆trsA* strain (2.4 kb). **(B)** Amplification of the hygromycin cassette, which has a size of 1.8 kb.
(S2_Fig.PDF)

S1 Table Strains used in this study. (S1_Table.PDF)

S2 Table Plasmids used in this study. (S2_Table.PDF)

S3 Table Oligonucleotides used in this study. (S3_Table.PDF)

S1 Movie Picking of *C. elegans* wild type. (S1_Movie.MP4)

S2 Movie Picking of *C. elegans* expressing *trsA.* (S2_Movie.MP4)
