# Supplementary material for: The Egh16-like virulence factor TrsA of the nematode-trapping fungus Arthrobotrys flagrans facilitates intrusion into its host Caenorhabditis elegans
Source: PLoS Pathog. 2025 Aug 25;21(8):e1013370. doi: 10.1371/journal.ppat.1013370 (PMC12377627; doi:10.1371/journal.ppat.1013370)
Supplement: S1 Fig — RNA was extracted from vegetative and nematode-induced mycelium. The expression was normalized to the A. flagrans actin gene (dfl_002353) (A) nipA (dfl_005407) is significantly upregulated in induced mycelium. Significance was determined using the unpaired two-tailed Student’s t-test (p-value = 0.0001; ****). (B) dfl_005403, dfl_005405 and dfl_005406 show no significant upregulation in nematode-induced mycelium. (S1_Fig.PDF) [file ppat.1013370.s001.pdf]

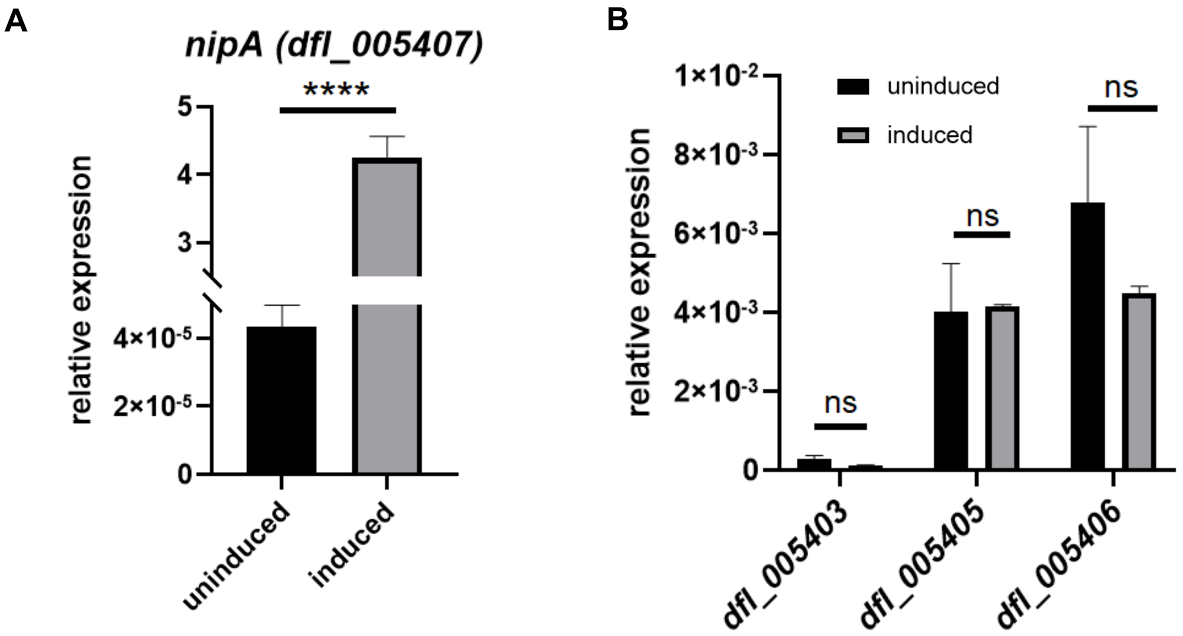

S1 Fig: RT-qPCR of the neighboring genes of *trsA*. RNA was extracted from vegetative and nematode-induced mycelium. The expression was normalized to the *A. flagrans* actin gene (*dfl_002353*) **(A)** *nipA* (*dfl_005407*) is significantly upregulated in induced mycelium. Significance was determined using the unpaired two-tailed Student's t-test (p-value = 0.0001; ****). **(B)** *dfl_005403*, *dfl_005405* and *dfl_005406* show no significant upregulation in nematode-induced mycelium.
