# Supplementary material for: The Egh16-like virulence factor TrsA of the nematode-trapping fungus Arthrobotrys flagrans facilitates intrusion into its host Caenorhabditis elegans
Source: PLoS Pathog. 2025 Aug 25;21(8):e1013370. doi: 10.1371/journal.ppat.1013370 (PMC12377627; doi:10.1371/journal.ppat.1013370)
Supplement: S2 Fig — (A) The upstream to downstream region of trsA was amplified in the wild type (2.1 kb) and the ∆trsA strain (2.4 kb). (B) Amplification of the hygromycin cassette, which has a size of 1.8 kb. (S2_Fig.PDF) [file ppat.1013370.s002.pdf]

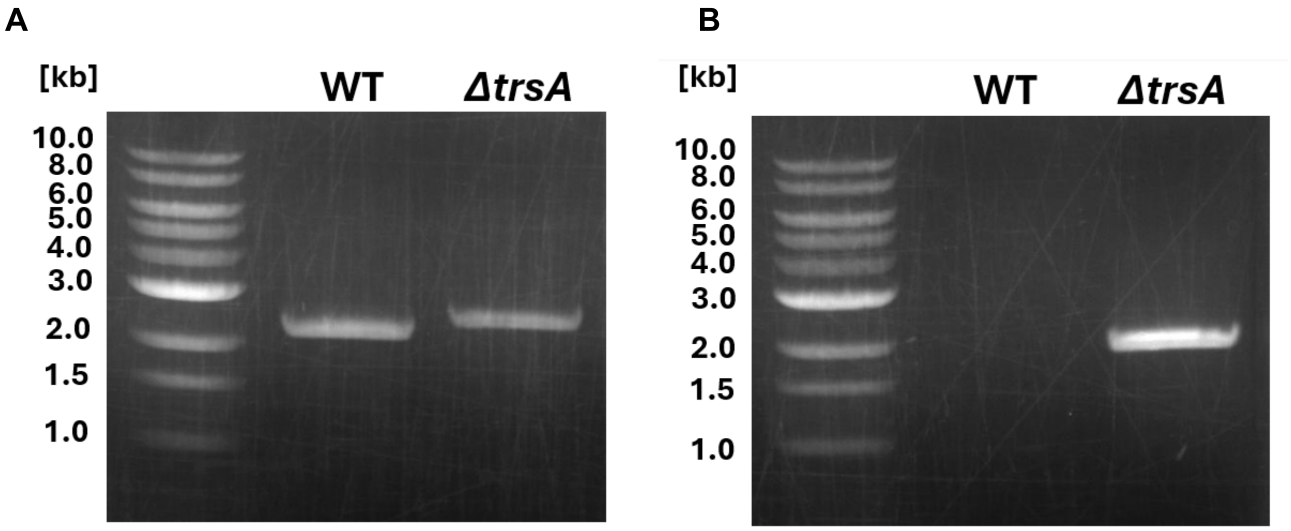

S2 Fig: PCR confirmation of the *trsA* deletion. (A) The upstream to downstream region of *trsA* was amplified in the wild type (2.1 kb) and the Δ*trsA* strain (2.4 kb). **(B)** Amplification of the hygromycin cassette, which has a size of 1.8 kb.
