# Supplementary material for: The Egh16-like virulence factor TrsA of the nematode-trapping fungus Arthrobotrys flagrans facilitates intrusion into its host Caenorhabditis elegans
Source: PLoS Pathog. 2025 Aug 25;21(8):e1013370. doi: 10.1371/journal.ppat.1013370 (PMC12377627; doi:10.1371/journal.ppat.1013370)
Supplement: S1 Table — (S1_Table.PDF) [file ppat.1013370.s003.pdf]

S1 Table: Strains used in this study.

Strain	Genotype	Reference
A. flagrans		
Wildtype	-	CBS-KNAW Fungal Biodiversity Centre, Utrecht
sJM47	Δ*trsA*, pJM28	This work
sEK01	sJM47 x *cyrA(p)::cyrA::mCherry*, pNH94	This work
sEK02	*gpdA(p)::trsA::LccC*, pEK02	This work
sEK03	*gpdA(p)::trsA*$^{\Delta SP}$*::LccC*, pEK03	This work
sEK06	sEK01 x *trsA(p)::trsA::gluC(t)*, pEK01	This work
sJM13	*trsA(p)::trsA::gfp*, pJM29	This work
sJM14	sVW10 x *trsA(p)::h2b::mCherry*, pJM30	This work
sJM43	*cyrA(p)::cyrA::mCherry::gluC(t)*, pNH94	[1]
sJM48	sJM13 x *nipA(p)::nipA::mCherry*, pJM16	This work
sVW10	*h2b(p)::h2b::GFP*, pVW04	[2]
C. elegans		
N2	Wildtype	University of Freiburg
KIT03	*hsp-16.48(p)::mScarlet,* pNH59	[3]
KIT46	*col-19(p)::mScarlet*, pJM72	[1]
KIT56	*col-19(p)::trsA::mScarlet*, pJM94	This work
KIT64	*hsp-16.48(p)::trsA*$^{\Delta SP}$*::mScarlet*, pEK10	This work
KIT65	*col-19(p)::trsA*$^{\Delta cys}$*::mScarlet*, pEK11	This work
KIT66	*eft-3(p)::trsA*$^{\Delta cys}$*::mScarlet*, pEK12	This work

KIT67	*hsp-16.48(p)::trsA::mScarlet*, pEK09	This work
KIT68	*hsp-16.48(p)::*$trsA^{his24/ala}$*::mScarlet,* pLES10	This work
E. coli		
TOP10	F- mcrA Δ(mrrhsdRMSmcrBC), φ80, lacZΔM15 ΔlacX74, nupG, recA1, araD139 Δ(araleu) 7697, galE15 galK16 rpsL(StrR) endA1 λ	Invitrogen, Karlsruhe
OP50	Ura^{-}	University of Freiburg
BL21 DE3	F- ompT hsdSB (rB- mB-) gal dcm (DE3)	Novagen, Darmstadt

References

1. Emser J, Wernet N, Hetzer B, Wohlmann E, Fischer R. The small cysteine-rich virulence factor NipA of *Arthrobotrys flagrans* interferes with cuticle integrity of *Caenorhabditis elegans*. Nat Commun. 2024;*15*(1):5795.
2. Wernet V, Wäckerle J, Fischer R. The STRIPAK component SipC is involved in morphology and cell-fate determination in the nematode-trapping fungus *Duddingtonia flagrans*. Genetics. 2022;220(1):iyab153.
3. Wernet N, Wernet V, Fischer R. The small-secreted cysteine-rich protein CyrA is a virulence factor of *Duddingtonia flagrans* during the *Caenorhabditis elegans* attack. PLoS Pathog. 2021;17(11):e1010028.
