# Supplementary material for: The Egh16-like virulence factor TrsA of the nematode-trapping fungus Arthrobotrys flagrans facilitates intrusion into its host Caenorhabditis elegans
Source: PLoS Pathog. 2025 Aug 25;21(8):e1013370. doi: 10.1371/journal.ppat.1013370 (PMC12377627; doi:10.1371/journal.ppat.1013370)
Supplement: S2 Table — (S2_Table.PDF) [file ppat.1013370.s004.pdf]

S2 Table: Plasmids used in this study

Name	Description	Reference
pEK01	*trsA(p)::trsA::gluC(t)*	This work
pEK02	*gpdA(p)::trsA::LccC*	This work
pEK03	*gpdA(p)::*$trsA^{\Delta SP}$*::LccC*	This work
pEK04	*eft-3(p)::trsA::mScarlet*	This work
pEK05	*eft-3(p)::*$trsA^{\Delta SP}$*::mScarlet*	This work
pEK09	*hsp-16.48(p)::trsA::mScarlet*	This work
pEK10	*hsp-16.48(p)::*$trsA^{\Delta SP}$*::mScarlet*	This work
pEK11	*col-19(p)::*$trsA^{\Delta cys}$*::mScarlet*	This work
pEK12	*eft-3(p)::*$trsA^{\Delta cys}$*::mScarlet*	This work
pJM10	*nipA(p)::h2b::mCherry::tubA(t)*	[1]
pJM16	*nipA(p)::nipA::mCherry*	[1]
pJM28	Δ*trsA* x pJet1.2	This work
pJM29	*trsA(p)::trsA::gfp*	This work
pJM30	*trsA(p)::h2b::mCherry*	This work
pJM72	*col-19(p)::mScarlet::unc-54 3'UTR*	[1]
pJM94	*col-19(p)::trsA::mScarlet::unc-54* 3'*UTR*	This work
pNH59	*hsp-16.48(p)::mScarlet::unc-54 3'UTR*	[2]
pNH94	*cyrA(p)::cyrA::mCherry::gluC(t)*	N. Wernet
pLES08	Cytiva™-tag::$trsA^{\Delta SP}$::his-tag::*T7(t)* from pDO10 in pET28a	This work
pLES10	*hsp-16.48(p)::*$trsA^{\Delta SP+his24/ala}$*::mScarlet::unc-54 3' UTR*	This work
pLES14	Cytiva™-tag::$trsA^{his24/ala}$::his-tag::*T7(t)*	This work

References

1. Emser J, Wernet N, Hetzer B, Wohlmann E, Fischer R. The small cysteine-rich virulence factor NipA of *Arthrobotrys flagrans* interferes with cuticle integrity of *Caenorhabditis elegans*. Nat Commun. 2024;*15*(1):5795.
2. Wernet N, Wernet V, Fischer R. The small-secreted cysteine-rich protein CyrA is a virulence factor of *Duddingtonia flagrans* during the *Caenorhabditis elegans* attack. PLoS Pathog. 2021;17(11):e1010028.
