# Supplementary material for: The Egh16-like virulence factor TrsA of the nematode-trapping fungus Arthrobotrys flagrans facilitates intrusion into its host Caenorhabditis elegans
Source: PLoS Pathog. 2025 Aug 25;21(8):e1013370. doi: 10.1371/journal.ppat.1013370 (PMC12377627; doi:10.1371/journal.ppat.1013370)
Supplement: S3 Table — (S3_Table.PDF) [file ppat.1013370.s005.pdf]

S3 Table. Oligonucleotides used in this study.

**Name**	**Sequence 5’→3’**	**Description**
trsA_gpdA(P)_OL_fw	TGAGCAGACATCACAGGCGCGCCATGCATTTCTCCACTCTTTTGAC	TrsA/laccase fusion with SP
trsA_LccC_OL_rev_neu	ATATAAGTTGAGATTTCATCGAGAAACCGGTTCTTGGGTCTTTGAGCA	TrsA/laccase fusion
trsA_noSP_gpdA(p)_OL_fwd	gagcagacatcacaGGCGCGCCATGCACTGTGTTTTTGTTGACGCC	TrsA/laccase fusion without SP
tubA(T)_rev	CAATTGGCTGTCTGGATCC	Recomplementation
trsA(P)_tubA(T)_OL_fwd	TCAATTGGCTGTCTGGATCCCTGCATATTTCCAACGCGCA	Recomplementation
trsA_gluc(T)_OL-rev	AGTTGAGATTTCATCGAGAATAGCGTATGTAGATAAGATGTATGAT	Recomplementation
tgluC_for	CGTATGTAGATAAGATGTATGATT	Recomplementation
5408_RT_fwd	TACACACCTGTTACCCCACC	RT-qPCR
5408_RT_rev	GAAGAGCCAAGACCCCTTCT	RT-qPCR
qpcr_hk_actin2_fwd	TCCAGACTGCCTCCCAGT	RT-qPCR
qpcr_hk_actin2_rev	AGGTCTTTTCTGACGTCGAC	RT-qPCR
RB_5408_pJet_OL_rev	ttgtaggagatcttctagaaagatGGGTGGCTATATATACACATTC	TrsA KO
RB_5408_trpCT_OL_fwd	AATGCTCTTTCCCTAAACTCCCCCCAAAGTCTTGGTGCACAAAAACTG	TrsA KO
LB_5408_trpCP_OL_rev	GTTGACCTCCACTAGCATTACACTTCTTGAAAGAGTGTCTCGGTTTT	TrsA KO
LB_5408_pJET_OL_fwd	cggatggctcgagtttttcagcaagatCTGCATATTTCCAACGCGCA	TrsA KO
5408_LB_trpCT_OL_fwd	AATGCTCTTTCCCTAAACTCCCCCCACTGCATATTTCCAACGCGCA	TrsA/GFP fusion

5408_LB_gfp_OL_rev	CCTCACCCTTGGAAACttaattaaTTCTCGATGAAATCTCAACTTATAT	TrsA/GFP fusion
Efi_bb_rev	GAATTCACTGGCCGTCGTTT	TrsA/GFP fusion
dfH2B_for	ATGCCACCAAAAGCCGCC	TrsA/GFP fusion
gfp_noATG_fwd	GTTTCCAAGGGTGAGGTAAG	TrsA/GFP fusion
p5408_pJM10_OL_fwd	TTGTAAAACGACGGCCAGTGAATTCGTCCTACAAATTACAGCAATGG	TrsA promoter/mCherry fusion
p5408_h2B_OL_rev	CTTTTCGGCGGCGGCTTTTGGTGGCATCTTGAAAGAGTGTCTCGGTTTT	TrsA promoter/mCherry fusion
h2b_cds_fwd	ATGCCACCAAAAGCCGCC	TrsA promoter/mCherry fusion
pJM10_rev	GAATTCACTGGCCGTCGTTT	TrsA promoter/mCherry fusion
5408_SB_for	AACTTCACAAGGAAACCTCGG	Southern blot analysis
5408_SB_rev	TGTCGTGCGTTGTCGTGTG	Southern blot analysis
TrsA_peft3_OL_fw	tttcagttgggaaacactttgctcATGCATTTCTCCACTCTTTTGACA	TrsA/mScarlet under *eft-3* promoter
TrsA_scarlet_OL_rev	CTCCCTTGCTGACCATACCGGTTTCTCGATGAAATCTCAACTTATATG	TrsA/mScarlet under *eft-3* promoter
trsA-noSP_peft3_OL_fw	ttttcagttgggaaacactttgctcATGCACTGTGTTTTTGTTGACGC	TrsA/mScarlet under *eft-3* promoter
trsA-noSP_scalet_OL_rev	TCCCTTGCTGACCATACCGGTTTCTCGATGAAATCTCAACTTATATG	TrsA/mScarlet under *eft-3* promoter
AgeI_scarlet_fwd	ACCGGTATGGTCAGCAAGG	TrsA/mScarlet under *eft-3* promoter
Peft3_rev	gagcaaagtgtttcccaact	TrsA/mScarlet under *eft-3* promoter
hs(p)-16.48_rev	ttcttgaagtttagagaatgaacag	Expression under *hsp-16.48* promoter
scarlet_fwd	ATGGTCAGCAAGGGAGAGG	Expression under *eft-3* promoter
peft3_trsA_OL_rev	AAAAGAGTGGAGAAATGCATgagcaaagtgtttcccaactg	Expression under *eft-3* promoter

trsA_hsp-16(P)_OL_fwd	tcattctctaaacttcaagaaATGCATTTCTCCACTCTTTTGACA	Expression under *eft-3* promoter
nipB_scar_OL_rev	CTCCCTTGCTGACCATACCGGTTTCTCGATGAAATCTCAACTTATATG	Expression under *eft-3, hsp-16.48* and *col-19* promoter
trsA_noSP_hsp-16(P)_OL_fwd	tcattctctaaacttcaagaaATGCACTGTGTTTTTGTTGACGC	Expression under *hsp-16.48* promoter
col-19(P)_trsA_OL_rev	AAAAGAGTGGAGAAATGCATgttgatgaactgatgtctttctaaatg	Expression under *col-19* promoter
trsA_mid_2_fwd	GAAGGAAGGGGCTTAAGACTG	Expression under *col-19* and *eft-3* promoter
TrsA_pDO10 _for	GGTCTGATTGCGAGCAACCATATGCACTGTGTTTTTGTTGACG	TrsA with n-terminal Cytiva™ Protein Select™ tag and c-terminal his-tag
TrsA_pET28a_no_stp_rev	TGGTGGTGGTGGTGGTGCTCGAGTTCTCGATGAAATCTCAACTTATATG	TrsA with n-terminal Cytiva™ Protein Select™ tag and c-terminal his-tag
hsp16.48_SP_rev	GGCGGATGTAACGTCGACA	Expression under *hsp-16.48* promoter with mutated his1
trsA_scarlet_rev	TGCCTCTCCCTTGCTGACCATTTCTCGATGAAATCTCAACTTATATG	Expression under *hsp-16.48* promoter with mutated his1
trsA_his1_mut_fw	ATTGTCGACGTTACATCCGCCGCCTGTGTTTTTGTTGACGCCTACG	Expression under *hsp-16.48* promoter with mutated his24
trsA_KO-check_fw	AGGGAGCTTATACTTACCGTAG	Analytic PCR
trsA_KO-check_rev	CTGTTATATAGGCACATGTCAC	Analytic PCR
TrpC_fw	AAGTGTAATGCTAGTGGAGGT	Analytic PCR
TrpC_rev	TGGGGGGAGTTTAGGGAAAG	Analytic PCR
TrsA_his24_mut_pdo_fw	GGTCTGATTGCGAGCAACCATATGGCCTGTGTTTTTGTTGACGCC	TrsA with mutated His24 with n-terminal Cytiva™ Protein Select™ tag and c-terminal his-tag
5403_RT_fwd	GATTCCAGCGGTTGTCCTTC	RT-qPCR

5403_RT_rev	GAATAGCCATTGCCCTCGTG	RT-qPCR
5405_RT_fwd	AGAGCTTAACGACCCAGCTT	RT-qPCR
5405_RT_rev	ACTGCTACACCAACATCCCA	RT-qPCR
5406_RT_fwd	CCGCATACAAAGATCGGAGC	RT-qPCR
5406_RT_rev	TCCGACGACGTTAAGTAGCA	RT-qPCR
5407_RT_for	ATGAAAGGCGCTATCCTCAT TAT	RT-qPCR
5407_RT_rev	CAATTGGCGGAATTCTTGCA TTT	RT-qPCR
